# MARCH E3 Ligases: Understudied Regulators of Pulmonary Immune Function

**DOI:** 10.70322/jrbtm.2026.10006

**Published:** 2026-08-12

**Authors:** Samuel Speaks, Anthony Pastore, MuChun Tsai

**Affiliations:** 1 Department of Microbial Infection and Immunity, The Ohio State University, Columbus, OH 43210, USA; 2 Division of Pulmonary, Critical Care, and Sleep Medicine, Department of Internal Medicine, The Ohio State University, Columbus, OH 43210, USA

**Keywords:** Membrane-associated RING-CH ligases, MARCH E3 ligases, E3 ubiquitin ligases, Ubiquitination

## Abstract

Membrane-associated RING-CH (MARCH) ligases are a family of 11 ubiquitin E3 ligases that regulate protein stability, trafficking, and signaling across diverse cellular contexts. Although MARCH ligases have been studied most extensively in immune regulation, antigen presentation, viral restriction, and cellular homeostasis, their roles in pulmonary biology remain incompletely defined. The lung is a highly specialized environmental interface that must preserve gas exchange while continuously responding to infectious, inflammatory, and sterile insults. These demands require careful regulation of epithelial and endothelial barrier integrity, innate and adaptive immune activation, and tissue repair. Current work suggests that MARCH ligases influence many of these processes by regulating inflammatory responses, cytokine receptors, antiviral signaling mediators, viral proteins, mitochondrial fission or fusion, junctional molecules, ciliary components, and remodeling pathways. In this review, we summarize current knowledge and highlight gaps in the understanding of MARCH ligase expression and function in lung-relevant cell types and disease contexts. Defining how MARCH ligases operate within specific lung compartments may reveal new regulatory mechanisms governing pulmonary immunity, barrier function, host defense, and lung remodeling.

## 1. Introduction

Ubiquitination is a fundamental post-translational modification in which ubiquitin, a 76-amino acid protein, is covalently attached to lysine residues on target proteins. While its most classical role is to label proteins for proteasomal degradation, ubiquitination also regulates protein stability, subcellular localization, trafficking, and interactions [1,2]. These diverse functions allow ubiquitination to shape nearly all aspects of homeostasis, including cell signaling, immune activation, and stress responses [3–8].

Ubiquitination is carried out by an enzymatic cascade involving three main components: E1 ubiquitin-activating enzymes (E1), E2 ubiquitin conjugating enzymes (E2), and E3 ubiquitin ligases (E3). The process begins when an ATP-dependent E1 activates ubiquitin to form a thioester bond [9]. Ubiquitin is then transferred to E2 [10], which collaborates with E3 to facilitate its attachment to a substrate protein [11]. Although the human genome encodes only two E1s and approximately 40 E2s, over 600 E3s have been identified, underscoring their critical role in conferring substrate specificity and regulatory diversity [12,13].

E3 ligases are broadly categorized into three major structural classes: homologous to E6-AP carboxy terminus (HECT), really interesting new gene (RING), and RING-between-RING (RBR). HECT and RBR ligases operate by forming a transient E3~ubiquitin thioester intermediate before transferring the ubiquitin to the substrate protein. In contrast, RING type ligases, by far the most abundant class, act to directly transfer a ubiquitin from E2 to the substrate protein without an intermediate [14]. Within the RING type class, the membrane associated RING-CH (MARCH) ligases form a distinct subfamily. These proteins are defined by a conserved C4HC3 zinc coordinating motif in their RING-CH domain, which structurally distinguishes them from the more common C3HC4 “RING-HC” motif found in most other RING ligases [15].

The human MARCH family consists of 11 E3 ligases (MARCH1–11), which were discovered due to their homology to viral proteins which downregulate major histone compatibility class I (MHC-I) receptors in Kaposi’s sarcoma-associated herpesvirus [16,17]. MARCH ligases are generally divided into two structural subgroups: canonical MARCHs (MARCH1–6, MARCH8–9, and MARCH11), which contain multiple transmembrane domains, and non-canonical cytosolic MARCHs (MARCH7 and MARCH10), which lack these membrane spanning regions [18]. Although the functions of these enzymes vary broadly, much work has focused on their role in immunity, including activities involved in antigen presentation, lymphocyte development, infection, and inflammation [18–22].

**Figure 1.**
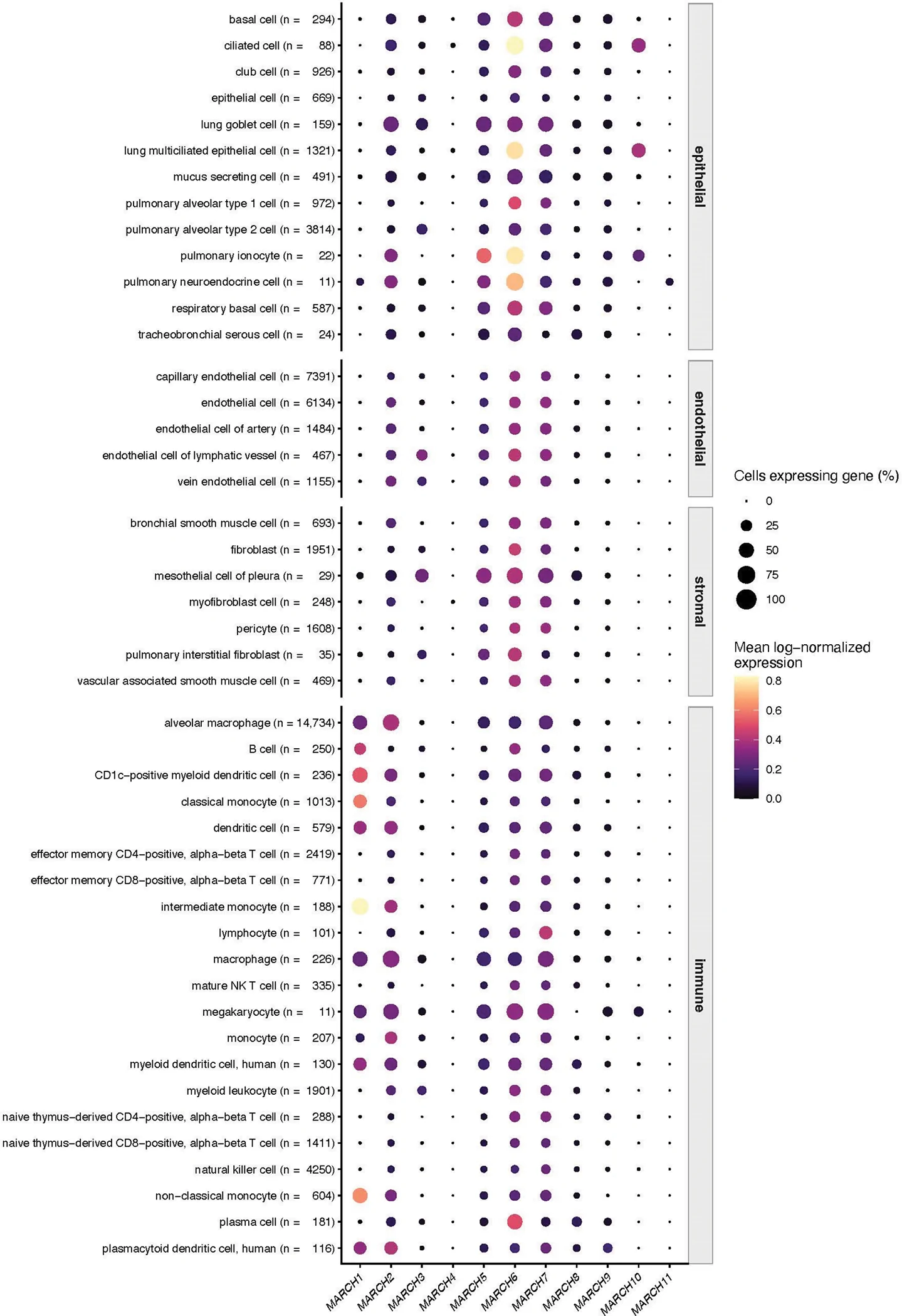
*MARCH*-family gene expression across cell types of the healthy human lung. Dot color represents mean log-normalized expression, and dot size represents the percentage of cells with detectable expression of each *MARCH* gene. Cell types are grouped by functional compartments, with number of analyzed cells shown in parentheses. Data was obtained from the Krasnow Lab Human Lung Atlas [24].

As the primary interface between the external environment and internal physiology, the lung is constantly exposed to inhaled pathogens, allergens, and particulate matter. Pulmonary homeostasis depends on coordinated interactions among four broad cellular compartments [23]. Epithelial cells form the airway and alveolar barriers and perform specialized functions, including mucociliary clearance and gas exchange; endothelial cells regulate vascular barrier integrity, perfusion, and leukocyte trafficking; stromal cells provide structural and extracellular matrix support; and resident and infiltrating immune cells mediate surveillance, pathogen clearance, and inflammatory responses. Within each of these major compartments, there is a large cellular diversity to carry out complex, coordinated functions and respond to stimuli. This diversity is showcased by single-cell RNA sequencing of healthy human lungs [24]. Our analysis of this publicly available dataset revealed broad *MARCH*-family gene expression across all four pulmonary compartments (Figure 1). Although the roles of MARCH ligases in immunity have been reviewed previously [18,20–22], their organ specific functions in the lung remain poorly understood. Thus, we aim to review what is currently known about MARCH ligase expression and function in the lung and identify key remaining questions for future investigation.

## 2. Materials and Methods

### Reanalysis of Single-Cell Lung Transcriptomic Data

A publicly available single-cell RNA-sequencing dataset from the Krasnow Lab Human Lung Cell Atlas was analyzed to characterize *MARCH*-family expression in healthy human lung cells [24]. The dataset comprises 65,662 cells from three donors. Analyses were restricted to the 60,993 cells annotated as originating from lung tissue; 4669 cells annotated as originating from the blood were excluded. No additional normalization, dimensionality reduction, or cell-type annotation was performed; published cell-type and compartment annotations were retained. As described by Travaglini et al. [24], 10× sequencing reads were processed using Cell Ranger version 2.0, and cells with fewer than 500 detected genes or 1000 unique molecular identifiers were excluded. Using Seurat version 2.3, UMI counts were normalized to 10,000 per cell and log-transformed using NormalizeData. Louvain clustering was performed using FindClusters with biologically informative principal components and empirically selected resolutions. Cells were subsequently separated into epithelial, endothelial, stromal, and immune compartments and iteratively re-clustered, as detailed by Travaglini et al. [24].

For each of the 11 *MARCH* genes and 46 annotated lung cell types, mean log-normalized expression was calculated across all cells assigned to that cell-type. The percentage of expressing cells was defined as the proportion with a normalized expression value greater than zero. Dot color represents the mean log-normalized expression on a common, unscaled scale, permitting comparisons among cell types and *MARCH* genes within the processed dataset; dot size represents the percentage of cells with detectable expression. The number of cells for each cell type is shown in the figure.

Data were imported and analyzed in R version 4.5.2 using anndataR version 1.0.2, SingleCellExperiment version 1.32.0, Matrix, and ggplot2 version 4.0.3. Analysis code is available upon request.

## 3. Main

### 3.1. MARCH-Family Expression Patterns

The lung is a highly complex organ composed of diverse cell types that work in concert to facilitate gas exchange between the external environment and the bloodstream. Broadly, lung-resident cells can be categorized into four functional compartments: epithelial, endothelial, stromal, and immune. Single cell sequencing data from healthy human lungs [24] show that nearly all *MARCH* E3 ligases are expressed in one or more of these compartments, suggesting diverse roles in maintaining pulmonary homeostasis (Figure 1).

Among the family, *MARCH2*, *MARCH5*, *MARCH6*, and *MARCH7* are the most highly expressed and widely distributed ligases. These are detected across nearly all major lung cell types, positioning them to have a broad functional impact across compartments. In addition, *MARCH8* and *MARCH9* show ubiquitous but lower-level expression present in many lung cell types, but at relatively lower levels than the above family members. While their expression is more subtle, their broad distribution still suggests a potential role in baseline cellular regulation.

In contrast, several *MARCH* ligases show cell type restricted expression patterns. For example, *MARCH1* is predominantly expressed in immune cells, particularly within the myeloid lineage. *MARCH10* is clearly enriched in ciliated epithelial cells, while *MARCH3* is modestly expressed across a few endothelial and stromal cell populations. Finally, *MARCH4* and *MARCH11* exhibit very low or undetectable expression across all cell types analyzed and will not be discussed further in this review.

### 3.2. MARCH-Family Proteins and Their Functions

#### 3.2.1. MARCH1

MARCH1 is primarily expressed in immune cells (Figure 1) and is best known for ubiquitinating and downregulating immunostimulatory molecules in antigen-presenting cells [25–27]. Classically, MARCH1 is induced by IL-10, leading to ubiquitination of key immunostimulatory molecules such as CD86 and MHC-II, on dendritic cells (DCs) and B cells [25–28]. Pulmonary pathogens are known to exploit this function to evade immune responses. For example, *Francisella tularensis* induces IL-10 expression, thereby upregulating MARCH1 and suppressing class II-mediated clearance [29].

From an immunological perspective, MARCH1 is critical to maintain a balance between inflammatory activation and tolerance. This equilibrium is most well studied in DCs, such that immature DCs express high levels of MARCH1 to limit premature T cell activation, whereas maturation is associated with MARCH1 downregulation and stability of immunostimulatory surface receptors [30,31]. However, the regulatory mechanisms of MARCH1 in other cell types are complex and remain incompletely understood, as B cells have distinct promoter elements compared with DCs [32]. Additionally, the regulation of MARCH1 by ubiquitination remains somewhat controversial. While some studies report that MARCH1 can undergo auto-ubiquitination, others have suggested that its turnover may rely on distinct ubiquitin ligases [28,33].

Beyond regulating host responses, MARCH1 can also influence microbial pathogenesis. In HIV-infected macrophages, MARCH1 reduces virion incorporation into the viral envelope [34,35]. In human cytomegalovirus infection, on the other hand, it enhances transferrin receptor expression, potentially increasing iron availability to support viral replication [36].

#### 3.2.2. MARCH2

MARCH2 is a ubiquitously expressed, membrane-associated E3 ubiquitin ligase. Indeed, MARCH2 is found in many pulmonary cell types, including various immune cells, vascular endothelial cells, and ionocytes (Figure 1). Although MARCH2 has not been greatly studied in the lung *in vivo*, it is known to regulate molecules critical for pulmonary homeostasis. For example, MARCH2 ubiquitinates ER-Golgi intermediate compartment protein 3, a cargo receptor of ⍺_1_-antitrypsin [37]. It also regulates the expression of cystic fibrosis transmembrane conductance regulator by associating with the CAL-syntaxin-6 complex at the Golgi apparatus and promoting CFTR ubiquitination [38,39].

Aside from regulating key pulmonary proteins, MARCH2 also contributes to the organization of cellular junctions and polarity in cellular systems. For example, MARCH2 is anchored to cell-cell junctions via DLG1 and can ubiquitinate DLG1, thereby linking MARCH2 to cellular polarity and junctional integrity [40]. This role in governing junctional stability was more explicitly shown in endothelial cells, where MARCH2 selectively targets VE-cadherin for ubiquitination via its transmembrane domain interactions, triggering degradation that results in adherens junction disruption [41].

Considering immunological contexts, MARCH2 is recognized to ubiquitinate I-kappa-B-epsilon (IKKε), an important antiviral type I interferon (IFN) signaling hub [42]. This is important in the context of negative-sense RNA virus infection, as IKK-mediated IFN signaling is critical for protection [43]. In addition to these homeostatic roles, MARCH2 participates in innate immune regulation. During negative-sense RNA virus infection, type I IFN signaling relies on IKKε, a direct substrate of MARCH2 [42]. By ubiquitinating IKKε and promoting its degradation, MARCH2 dampens IFN responses, a pathway that viruses may exploit to weaken host defenses in the respiratory tract [43].

#### 3.2.3. MARCH3

MARCH3 has not been well-characterized overall within the lung, but the Human Protein Atlas identifies MARCH3 to be expressed predominantly in endothelial cells along with various immune cells, including mast cells, monocytes, T-cells, and B-cells, throughout multiple organs in the human body. Although MARCH3 appears to be predominantly found in fibroblasts and endothelial cells (Figure 1), there are no works that currently describe MARCH3 function specifically in the lungs. However, in human vein and cerebral microvascular endothelial cells, MARCH3 has been suggested to be involved with endothelial barrier function with strengthened tight junctions when MARCH3 is depleted [44]. Regarding its immune function, it has been described to target IL-1 receptor and IL-3 receptor α-chain for polyubiquitination to regulate the IL-1 triggered inflammatory response and IL-3, respectively [45,46]. In conjunction with MARCH2, MARCH3 has also been found to target IL-5Rα for polyubiquitination and lysosomal degradation and may play a role in the negative regulation of IL-5 triggered eosinophil maturation and eosinophilic airway inflammation [47]. Although not described in the lungs, MARCH3 is reported to mediate ubiquitination and degradation of the inflammasome component NLRP3 in rat peritoneal macrophages, thereby reducing NLRP3-mediated macrophage pyroptosis [48]. It has also been reported to mediate polyubiquitination and lysosomal degradation of IL-6Rα, thereby regulating colitis-associated carcinogenesis [49]. Furthermore, in Zika infection, MARCH3 is described to downregulate a T-cell immunoglobulin mucin family member-1 (TIM-1), which is a transmembrane glycoprotein reported to act as an entry receptor for various flaviviruses [50]. Along with MARCH2, MARCH3 polyubiquitinates TIM-1, targeting it for proteasomal degradation [50].

#### 3.2.4. MARCH5

MARCH5 is ubiquitously expressed, with the highest expression in B cells and lung epithelium, particularly in pulmonary ionocytes (Figure 1). The main role of MARCH5 is to regulate proteins involved in mitochondrial fission and fusion [51]. Depletion of MARCH5 leads to mitochondrial fragmentation through its interaction with mitochondrial dynamic protein of 49 kDa (MiD49), in which MARCH5 marks dynamin-related protein 1 for degradation [51]. While MARCH5 is highly expressed in the pulmonary ionocytes, little is known about its function within the lung [52]. MARCH5 is known to regulate mitochondrial function through the clearance of mitochondrial fusion protein Dynamin-related protein 1 (Drp1) by proteasomal degradation [53]. In the case of MARCH5 depletion, Drp1 is no longer degraded, and uncontrollable mitochondrial division occurs [54]. Further, MARCH5 degrades misfolded proteins, namely mutated Superoxide Dismutase 1 (SOD-1), which is known to increase reactive oxygen species concentration within the mitochondria [55]. However, none of these functions were tested in pulmonary models.

In immune contexts, MARCH5 is involved in innate response pathways critical to pulmonary pathogen defense. MARCH5 is a known regulator of Mitochondrial anti-viral signaling (MAVS) protein [56], an important antiviral signaling hub, by ubiquitinating K7 and K500, thereby promoting proteasomal degradation. In an influenza infection model, MARCH5 knockdown cells produce more type I IFN, which inhibits viral replication compared to control cells [56].

MARCH5 also regulates NF-κB, a central hub of inflammatory gene expression, through its interaction with TRAF family member-associated NF-κB activator (TANK) [57,58]. TANK is a TRAF-binding adaptor that normally restrains TRAF-dependent signaling, including TRAF6-mediated NF-κB activation downstream of innate immune receptors. MARCH5 polyubiquitinates TANK, impairing this inhibitory function and thereby relieving a brake on TRAF6-driven NF-κB signaling [57,59].

Within the lung endothelium, MARCH5 serves as a protective factor during hypoxia due to its role in the Akt/eNOS pathway. MARCH5 is significantly decreased during ischemic injury in endothelial cells, which compromises endothelial cell function. MARCH5 depletion shows impaired proliferation, angiogenesis, and migration, which was ameliorated by exogenous rescue. Mechanistic studies revealed that MARCH5 depletion disrupts eNOS, p-eNOS, and Akt1-mediated angiogenesis [60,61], highlighting its protective role during hypoxic lung injury.

#### 3.2.5. MARCH6

MARCH6 is expressed ubiquitously throughout the body, as it is a polytropic membrane protein integrated into the endoplasmic reticulum membrane. MARCH6 mediates cholesterol synthesis and degradation, as well as endoplasmic reticulum associated degradation, through autoubiquitination [62]. No work currently exists describing MARCH6’s role in pulmonary homeostasis. However, it is a known inhibitor of angiogenesis in umbilical vein endothelial cells [63], highlighting its potential importance in pulmonary development and disease. MARCH6 represents a currently understudied and potentially important ligase within the pulmonary microenvironment, warranting future investigation.

#### 3.2.6. MARCH7

MARCH7, also known as Axotrophin, is ubiquitously expressed in the lung throughout stromal, epithelial, endothelial, and immune cell compartments. Its expression is tightly regulated by deubiquitylating enzymes USP7 within cellular nuclei, and USP9x, which acts in the cytosol [64]. Although MARCH7 has not been explicitly studied within lung tissue, cellular studies have shown its importance for fundamental pulmonary processes. For example, MARCH7 ubiquitinates and degrades nephrocystin-5, a positive regulator of ciliogenesis, which is carefully regulated to control cilia formation at distinct points within the cell cycle [65]. Furthermore, MARCH7 also controls NLRP3 ubiquitination [66,67], a well-described innate immune sensor responsible for detecting and coordinating responses against a plethora of pulmonary pathogens, most recently Influenza A virus [68].

Regarding MARCH7’s broader role in immunity, it is most well-known to regulate immunological tolerance. Initial gene expression profiling identified it as selectively upregulated in tolerant splenocytes, in a murine model of transplantation [69]. Subsequent functional studies using MARCH7 deficient mice demonstrated that loss of Axotrophin results in enhanced T cell proliferation and increased production of leukemia inhibitory factor, reinforcing MARCH7s role in regulating the balance between tolerance and rejection [70]. These findings were further supported by human studies correlating Axotrophin and FOXP3 expression with CD4^+^CD25^+^ T-regulatory cells [71], solidifying MARCH7 as a key regulator of immunological tolerance and activity and emphasizing the need for further investigation into its pulmonary specific roles.

#### 3.2.7. MARCH8

MARCH8 is expressed at relatively low but ubiquitous levels in the lung, with detectable expression in stromal, immune, epithelial, and endothelial cells (Figure 1). Subcellularly, it localizes primarily to intracellular membrane compartments, including the Golgi apparatus, endosomes, and lysosomes.

Emerging evidence implicates MARCH8 in multiple forms of pulmonary pathology. In fibrotic lung disease, MARCH8 expression is reduced in patient tissue samples. Experimental manipulation demonstrates an inverse relationship between MARCH8 levels and fibrotic markers including ⍺-Smooth Muscle Actin, collagen type I, and fibronectin, suggesting MARCH8 may act as a negative regulator of fibrotic remodeling [72]. Similarly, transcriptome analysis in non-small cell lung cancer (NSCLC) shows that reduced MARCH8 expression correlates with more advanced disease and poorer prognosis. Mechanistically, MARCH8 promotes degradation of oncogenic signaling mediators. Overexpression of MARCH8 decreases drivers of cellular proliferation, such as phosphorylated AKT and mTOR, and suppresses metastatic markers such as N-cadherin, Snail, and Twist [73]. In addition to its endogenous tumor-suppressive role, MARCH8 has been implicated in EGFR-targeted therapy responses. Treatment with the EGFR inhibitor Osimertinib induces MARCH8-dependent degradation of Death Receptor 4 (DR4) and reduces DR4 transcription, effects associated with improved survival [74]. Collectively, these findings position MARCH8 as a multifaceted regulator of pulmonary disease progression.

Beyond its role in pulmonary pathology, MARCH8 is well characterized as a regulator of antiviral host defense. Most studies have focused on its ability to restrict viruses, even respiratory viruses such as Influenza A virus (IAV), Respiratory Syncytial Virus (RSV), and Severe Acute Respiratory Syndrome Coronavirus 2 (SARS-CoV-2). In IAV infection, MARCH8 limits viral propagation through at least two distinct mechanisms: (1) it interferes with hemagglutinin (HA) cleavage and maturation by furin [75], and (2) it ubiquitinates the viral M2 protein, promoting its degradation [76]. Together, these mechanisms reduce the production of infectious progeny. In RSV, MARCH8 targets the viral small hydrophobic (SH) protein for degradation, thereby suppressing host cell apoptosis and permitting productive replication. Loss of SH in the presence of MARCH8 enhances apoptosis and limits viral spread [77]. More recently, MARCH8 has been shown to ubiquitinate both the nucleocapsid (N) and spike (S) proteins of SARS-CoV-2, further supporting a model in which MARCH8 restricts viral replication through direct targeting of essential viral proteins [78,79]. In addition to directly modifying viral membrane glycoproteins, MARCH8 also regulates the early stages of infection by modulating host restriction factors. Notably, MARCH8 influences the expression of Interferon-Induced Transmembrane Protein-3 (IFITM3) [80], a well-established antiviral factor that restricts entry of multiple respiratory viruses, including IAV [81,82], RSV [83], SARS-CoV-2 [84,85], human metapneumovirus [86], and more. Collectively, these findings indicate that MARCH8 restricts respiratory viruses through dual mechanisms: direct ubiquitination of viral components and indirect amplification of host antiviral defenses.

In addition to its direct antiviral functions, MARCH8 serves as a negative regulator of innate and adaptive immune signaling, acting as a molecular rheostat to control inflammation. Within RNA sensing pathways, MARCH8 is recruited by Tetherin to ubiquitinate the mitochondrial antiviral signaling protein (MAVS), promoting its degradation and dampening downstream IFN production following RIG-I-like receptor (RLR) activation [87,88]. This mechanism provides feedback inhibition to limit hyperinflammatory responses after RNA detection. MARCH8 similarly regulates DNA sensing pathways by ubiquitinating cyclic GMP-AMP synthase (cGAS) following DNA sensing, thereby restraining type I IFN production [89]. Beyond innate sensing pathways, MARCH8 also modulates adaptive immune signaling. It ubiquitinates the IL-7 receptor alpha chain (IL-7Ra), attenuating IL-7 signaling, which is critical for T cell homeostasis and expansion [90]. Through this mechanism, MARCH8 contributes to the regulation of T cell production and helps limit autoimmune pathology. Together, these findings position MARCH8 as a key checkpoint that balances antiviral defense with prevention of immune-based tissue damage.

#### 3.2.8. MARCH9

MARCH9, another under-characterized E3 ligase, has been reported in the lungs to decrease intercellular adhesion molecule 1 (ICAM-1), a protein that plays a role in cell migration [91]. MARCH9 has been implicated in its involvement with the downregulation of cell surface major histocompatibility complex class I (MHC-I) by endosomal targeting and degradation [92] or in MHC-I trafficking to endosomes by ubiquitinating various proteins, such as CD1a and XPT [93]. MARCH9 appears to be localized in the trans Golgi network, where MARCH9 may also involve maintaining Golgi integrity [92–94]. Further, in conjunction with MARCH4, MARCH9 has been described to downregulate Mult1, a ligand to the stimulatory receptor NKG2D expressed by NK cells and some T cell subsets, and MHC-I [92,95]. MARCH9 is also involved with inflammatory processes, inhibiting the inflammasome NLRP3 activation by facilitating proteasomal degradation of NLRP3 [96]. MARCH9 also demonstrated a significant reduction in IL-6 responsiveness and a significant decrease in cell-surface IL-6Rα in the mouse M1 myeloid leukemia cell line [91]. Further studies are necessary to investigate the mechanisms involved in the lungs.

#### 3.2.9. MARCH10

Within the lungs, MARCH10 is predominantly expressed within ciliated epithelial cells, where it plays a critical role in maintaining ciliary function during viral disease [97]. Influenza A infection has been shown to decrease MARCH10 expression in ciliated lung epithelial cells and reduce ciliary beating. Studies revealed that knock down of MARCH10 in ciliated epithelial cells decreased dynein axonemal intermediate chain 1 (DNAI1), a critical component of cilia [97]. While not expressed within immune cells, MARCH10 plays a role in the innate immune response to viral infection, namely influenza A virus. During influenza virus infection, viral envelope glycoprotein hemagglutinin (HA) binds exposed sialic acid residues that are abundant on lung epithelial cells to trigger endocytosis and subsequent fusion [98]. MARCH10 has been shown to polyubiquitinate influenza HA, targeting it for degradation [99]. MARCH10 overexpression in lung epithelial cells infected with influenza virus infection also decreased influenza HA protein levels and decreased IL-1β cytokine expression [97,98].

## 4. Discussion

This review highlights the MARCH family as an understudied group of E3 ligases with broad relevance to pulmonary biology (Table 1). Reanalysis of healthy human lung single-cell data suggests that nearly all MARCH ligases are expressed in one or more lung compartments, with some broadly distributed and others restricted to specific cell populations (Figure 1). Together with available functional studies, these expression patterns suggest that MARCH ligases may coordinate a variety of pulmonary functions, including immune balance, epithelial and endothelial homeostasis, antiviral defense, and tissue remodeling.

### 4.1. MARCH E3 Ligases Serve as Critical Immune Modulators

MARCH E3 ligases function as pulmonary immune modulators by tuning the abundance, localization, and signaling activity of proteins that govern host defense. Rather than acting solely as pro- or anti-inflammatory factors, MARCH ligases appear to balance the threshold between protective immunity and tissue-damaging inflammation. In adaptive immunity, family members such as MARCH1 and MARCH8 regulate antigen presentation and co-stimulatory molecule availability, thereby shaping T-cell activation and tolerance [25–28,30,31,100]. In innate immunity, MARCH3, MARCH7, and MARCH9 have been linked to regulation of the NLRP3 inflammasome, a central inflammatory sensor that coordinates IL-1β-driven host defense but can also promote hyperinflammatory lung injury [68,101–104]. Other MARCH ligases modulate broader cytokine and antiviral signaling programs, including pathways controlled by NF-κB, IKKε, MAVS, and cGAS-STING [42,43,56–58,87–89]. These activities position MARCH2, MARCH5, MARCH8, and related family members at key intersections between inflammatory activation, IFN signaling, and immune resolution. Together, these studies support a model in which MARCH ligases preserve pulmonary immune homeostasis by restraining excessive inflammation while maintaining sufficient antimicrobial and antiviral defense.

### 4.2. MARCH-Mediated Antiviral Activity Is Paradoxical and Highly Context Dependent

Respiratory viral infection represents one of the most clinically relevant pulmonary insults, yet the viral-focused MARCH literature often emphasizes MARCH proteins as negative regulators of antiviral immunity. In this framework, MARCH family members restrain excessive IFN signaling by targeting key innate immune mediators, including MAVS, cGAS, IKKε, and related signaling complexes [42,43,56–58,87–89], thereby limiting the magnitude and duration of host inflammatory responses. This regulatory function may protect against IFN-driven immunopathology, but it can also create a permissive environment for viral replication depending on the timing, cell type, and pathogen involved. Conversely, several MARCH proteins function as direct antiviral restriction factors. MARCH8, for example, can target viral structural or accessory proteins, including influenza A virus M2, respiratory syncytial virus SH, and SARSCoV-2 nucleocapsid-associated pathways, thereby impairing productive viral replication or release [75–79]. MARCH-dependent antiviral defense may also occur indirectly at the epithelial barrier; MARCH10 supports ciliary function, and its loss during influenza infection may compromise mucociliary clearance [97,99]. Thus, although MARCH ligases are frequently positioned as immunoregulatory factors that blunt antiviral signaling, their net effect during pulmonary viral infection is likely determined by the balance between host-signal attenuation, direct viral protein restriction, and preservation of airway epithelial defense.

### 4.3. Barrier Maintenance and Integrity Remain Underexpored Functions of MARCH E3 Ligases in the Lung

The pulmonary alveolar-capillary interface requires tightly regulated epithelial and endothelial permeability to preserve gas exchange while permitting immune surveillance, fluid balance, and tissue repair. Although few studies have directly examined MARCH ligases at the pulmonary air-blood barrier, emerging evidence from epithelial and endothelial systems suggests that several family members may regulate junctional stability, vascular permeability, and mucosal defense. MARCH2, for example, has been implicated in the control of endothelial cell-cell adhesion by regulating VE-cadherin, a central determinant of vascular barrier integrity and paracellular permeability [41]. Similarly, MARCH3 and MARCH6 have been shown in non-pulmonary endothelial models to weaken venous or microvascular barrier function, raising the possibility that analogous mechanisms may contribute to pulmonary vascular leak during acute lung injury, viral pneumonia, sepsis, or ARDS [44,63]. MARCH5 may also be relevant in this context because its role in angiogenic signaling could influence vascular remodeling in diseases characterized by endothelial dysfunction, including pulmonary hypertension, fibrotic lung disease, and post-injury repair. In the airway epithelium, MARCH10 supports ciliary structure and function, linking this family to mucociliary clearance, pathogen removal, and epithelial host defense [97]. Together, these findings position MARCH ligases as potential regulators of pulmonary barrier homeostasis across endothelial, epithelial, and ciliated cell compartments. Defining their lung-specific effects may reveal an underappreciated role for MARCH proteins in preserving alveolar-capillary integrity and limiting barrier failure during pulmonary injury.

### 4.4. Chronic Remodeling, Fibrosis, and Malignancy Remain Underexplored Consequences of MARCH Ligase Biology

Although MARCH ligases are most often discussed in the context of acute immune regulation, antiviral defense, and protein trafficking, emerging evidence suggests that this family may also influence longer-term programs of tissue remodeling. This concept is best developed for MARCH8. In fibrotic lung disease, MARCH8 expression is reduced in idiopathic pulmonary fibrosis and in experimental fibrosis models, and loss of MARCH8 promotes the fibroblast-to-myofibroblast transition with increased expression of profibrotic markers such as α-SMA, collagen I, and fibronectin [72]. In parallel, MARCH8 appears relevant to malignant remodeling in non-small cell lung cancer, where reduced expression has been associated with more aggressive disease features and poorer prognosis [73]. Mechanistically, MARCH8 has been linked to the regulation of PI3K-AKT-mTOR signaling, epithelial-to-mesenchymal transition, and EGFR-targeted therapy biology through the proteasomal degradation of death receptor 4 [73,74]. While these studies are concentrated largely on MARCH8, they raise a broader possibility: MARCH ligases may shape not only acute inflammatory responses but also the chronic repair, fibrotic remodeling, and malignant transformation programs that follow persistent or unresolved lung injury.

### 4.5. Future Directions in Pulmonary MARCH Biology

Current MARCH E3 ligase literature has identified multiple candidate pathways through which this family may regulate pulmonary immunity, barrier function, antiviral defense, and tissue remodeling. However, a major barrier to defining their role in lung biology is the limited validation of these mechanisms in primary lung cells, compartment-specific systems, and *in vivo* pulmonary disease models. Future studies should determine how individual MARCH ligases function across epithelial, endothelial, stromal, and immune compartments during homeostasis, acute injury, infection, and chronic remodeling. In addition, the potential for functional redundancy or compensation among MARCH family members remains largely unexplored and may be essential for understanding their context-dependent effects. As host-directed mechanisms of inflammatory, infectious, and fibrotic lung disease continue to emerge, the MARCH family represents an important but still understudied regulatory layer in pulmonary biology.

## Figures and Tables

**Table 1.** Core attributes of MARCH E3 ligases in pulmonary biology.

Gene	Localization	Function	Synergistic Interactions	References
*MARCH1*	Immune	Downregulation of immunostimulatory molecules in antigen-presenting cells Ubiquitination of MHC-II and CD86		[25–28]
*MARCH2*	Ubiquitous	Trafficking secretory proteins via ubiquitination of ERGIC3 Promotes CFTR ubiquitination Organization of cellular junctions via DLG1 IKKε Regulation	Polyubiquitination of IL-5Rα, TIM-1 in conjunction with MARCH3	[38-42,47,50]
*MARCH3*	Endothelial, immune, fibroblasts	Endothelial barrier stability Regulation of IL-1, IL-3 receptors Ubiquitination of IL-6Rα	Polyubiquitination of IL-5Rα, TIM-1 in conjunction with MARCH2	[44-50,66,67]
*MARCH5*	Pulmonary ionocytes, immune, epithelial, endothelial	Mitochondrial stability via Drp1 Misfolded protein clearance Regulation of MAVS, NF-κB		[51–61]
*MARCH6*	Ubiquitous	Cholesterol synthesis		[62,63]
*MARCH7*	Ubiquitous	Ciliogenesis T cell proliferation		[65–67,71]
*MARCH8*	Low but ubiquitous	Regulator of fibrotic remodeling. NSCLC, cellular proliferation Antigen Presentation Antiviral host defense via IFIT3 DNA/RNA Sensing Ubiquitination of viral protein M2		[72,76-86,89]
*MARCH9*	Low but ubiquitous	Cellular migration MHC-I downregulation Golgi integrity NK/T cell stimulation regulation Inflammatory regulation	Downregulation of Mult1 in conjunction with MARCH4	[91–96]
*MARCH10*	Epithelial	Ciliary beating Ubiquitination of influenza virus protein HA		[97–99]

## Data Availability

Single cell sequencing data is publicly available [24]. Code available on request.
